# Combination of Blood Routine Examination and T-SPOT.TB Assay for Distinguishing Between Active Tuberculosis and Latent Tuberculosis Infection

**DOI:** 10.3389/fcimb.2021.575650

**Published:** 2021-06-29

**Authors:** Ying Luo, Guoxing Tang, Xu Yuan, Qun Lin, Liyan Mao, Huijuan Song, Ying Xue, Shiji Wu, Renren Ouyang, Hongyan Hou, Feng Wang, Ziyong Sun

**Affiliations:** ^1^ Department of Laboratory Medicine, Tongji Hospital, Tongji Medical College, Huazhong University of Science and Technology, Wuhan, China; ^2^ Department of Immunology, School of Basic Medicine, Tongji Medical College, Huazhong University of Science and Technology, Wuhan, China; ^3^ Department of Clinical Immunology, Tongji Hospital, Tongji Medical College, Huazhong University of Sciences and Technology, Wuhan, China

**Keywords:** active tuberculosis, latent tuberculosis infection, differential diagnosis, diagnostic model, blood routine examination, T-SPOT.TB

## Abstract

**Background:**

Distinguishing between active tuberculosis (ATB) and latent tuberculosis infection (LTBI) remains challenging.

**Methods:**

Between 2013 and 2019, 2,059 (1,097 ATB and 962 LTBI) and another 883 (372 ATB and 511 LTBI) participants were recruited based on positive T-SPOT.TB (T-SPOT) results from Qiaokou (training) and Caidian (validation) cohorts, respectively. Blood routine examination (BRE) was performed simultaneously. Diagnostic model was established according to multivariate logistic regression.

**Results:**

Significant differences were observed in all indicators of BRE and T-SPOT assay between ATB and LTBI. Diagnostic model built on BRE showed area under the curve (AUC) of 0.846 and 0.850 for discriminating ATB from LTBI in the training and validation cohorts, respectively. Meanwhile, TB-specific antigens spot-forming cells (SFC) (the larger of early secreted antigenic target 6 and culture filtrate protein 10 SFC in T-SPOT assay) produced lower AUC of 0.775 and 0.800 in the training and validation cohorts, respectively. The diagnostic model based on combination of BRE and T-SPOT showed an AUC of 0.909 for differentiating ATB from LTBI, with 78.03% sensitivity and 90.23% specificity when a cutoff value of 0.587 was used in the training cohort. Application of the model to the validation cohort showed similar performance. The AUC, sensitivity, and specificity were 0.910, 78.23%, and 90.02%, respectively. Furthermore, we also assessed the performance of our model in differentiating ATB from LTBI with lung lesions. Receiver operating characteristic analysis showed that the AUC of established model was 0.885, while a threshold of 0.587 yield a sensitivity of 78.03% and a specificity of 85.69%, respectively.

**Conclusions:**

The diagnostic model based on combination of BRE and T-SPOT could provide a reliable differentiation between ATB and LTBI.

## Introduction

Tuberculosis (TB), caused by *Mycobacterium tuberculosis* (Mtb) infection, remains a major health burden worldwide (Furin et al., 2019). Globally, an estimated 10 million people fell ill with TB in 2018 (World Health Organization, 2019). Meanwhile, approximately a quarter of the world’s population has latent tuberculosis infection (LTBI), and most of them remain asymptomatic, with 5–10% developing active tuberculosis (ATB) (Blumberg and Ernst, 2016; Cohen et al., 2019; World Health Organization, 2019). Hence, the development of differential diagnosis between ATB and LTBI is a vital goal for TB management and control (Furin et al., 2019).

Diagnosis of ATB currently relies on microbiologic tests such as smear microscopy, culture, and molecular assays such as Xpert MTB/RIF. However, these tests are either time-consuming or have unsatisfactory sensitivities (World Health Organization, 2015; Horne et al., 2019). Although the recently developed Xpert MTB/RIF Ultra showed an increase in sensitivity, it still cannot meet clinical requirements (Chakravorty et al., 2017; Dorman et al., 2018; Horne et al., 2019). Besides, interferon-gamma release assays including T-SPOT.TB (T-SPOT) and QuantiFERON-TB Gold In-Tube (QFT-GIT) are used to diagnose TB infection, but both of them are not able to distinguish ATB from LTBI (Turetz and Ma, 2016). In addition, although many immunological markers were also evaluated for overcoming the problem (Adekambi et al., 2015; Won et al., 2017; Musvosvi et al., 2018; Roy Chowdhury et al., 2018), the corresponding detection is costly and infeasible, such as the need for special instrument like flow cytometer. Several recent studies have explored new approaches for this target, including transcriptome (Singhania et al., 2018; Roe et al., 2019), metabolome (Weiner et al., 2018; Dai et al., 2019), proteome (Chaisson et al., 2019), and genome (Suliman et al., 2018). However, these methods were exploratory and lacked simplicity. Thus, more sensitive and specific assays that are faster and lower cost would be a great advance for the field.

Blood routine examination (BRE) is the most common test performed in clinical practice. The potential use of BRE for TB diagnostic purpose has rarely been previously reported. The present study investigated indicators of BRE and T-SPOT in individuals with ATB and LTBI. A diagnostic model based on combination of various indicators was established for differential diagnosis between these two conditions.

## Materials and Methods

### Study Design

This study was carried out at Tongji Hospital (Qiaokou cohort, the largest hospital in central China) and Sino-French New City Hospital (Caidian cohort, a branch hospital of Tongji Hospital). Subjects in Qiaokou cohort were enrolled between January 2013 and October 2019; and those in Caidian cohort were enrolled from January 2017 to October 2019. All participants were recruited based on positive T-SPOT results. BRE was performed in all subjects simultaneously. ATB was diagnosed as having positive results of Xpert MTB/RIF, and/or Mtb culture (Mycobacterial Growth Indicator Tube 960 and Lowenstein-Jensen media) in sputum, bronchoalveolar lavage fluid, or biopsy tissue, with clinical symptoms and radiological characteristics suggestive of TB. Individuals with positive T-SPOT results but without clinical or radiographic evidence of ATB were defined as LTBI. The exclusion criteria were as follows: (1) patients younger than 17 years of age and (2) patients undergoing anti-TB treatment. This study was approved by the Ethics Committee of Tongji Hospital, Tongji Medical College, Huazhong University of Science and Technology, Wuhan, China.

### BRE

Ethylenediaminetetraacetic acid-anticoagulated peripheral blood samples were collected from participants and BRE was performed using XN-9000 Sysmex (Sysmex Co., Kobe, Japan) according to the manufacturer’s instructions. The indicators obtained were as following: white blood cell count (WBC#), neutrophil percentage (NEUT%), neutrophil count (NEUT#), lymphocyte percentage (LYMPH%), lymphocyte count (LYMPH#), monocyte percentage (MONO%), monocyte count (MONO#), eosinophil percentage (EO%), eosinophil count (EO#), basophil percentage (BASO%), basophil count (BASO#), red blood cell count (RBC#), hemoglobin (HGB), hematocrit (HCT), mean corpuscular volume (MCV), mean corpuscular hemoglobin (MCH), mean corpuscular hemoglobin concentration (MCHC), coefficient variation of red blood cell volume distribution width (RDW-CV), standard deviation in red cell distribution width (RDW-SD), platelet count (PLT#), platelet distribution width (PDW), mean platelet volume (MPV), platelet larger cell ratio (PLCR), thrombocytocrit (PCT).

### T-SPOT Assay

Samples of heparinized peripheral blood were collected and were analyzed using T-SPOT assay (Oxford Immunotec, Oxford, UK) according to the manufacturer’s instructions. There are four indicators in the results of T-SPOT assay: negative control spot-forming cells (SFC), early secreted antigenic target 6 (ESAT-6) SFC, culture filtrate protein 10 (CFP-10) SFC, and positive control SFC. The larger of ESAT-6 SFC and CFP-10 SFC was defined as TB-specific antigens (TBAg) SFC.

### Statistical Analysis

Differences between ATB and LTBI groups were compared using Mann-Whitney *U* test, or chi-square test. To build the diagnostic model for differentiating ATB from LTBI, all variables with statistical significance were taken as candidates for further multivariable logistic regression analyses; and then the regression equation (diagnostic model) was obtained and a score for each individual was calculated.

Receiver operating characteristic (ROC) analysis was performed to test the ability of various methods to distinguish ATB from LTBI. Area under the curve (AUC), sensitivity, specificity, positive predictive value (PPV), negative predictive value (NPV), positive likelihood ratio (PLR), negative likelihood ratio (NLR), and accuracy, together with their 95% confidence intervals (CI), were calculated. Statistical analysis was performed using SPSS 25.0 (SPSS, Chicago, IL, USA) and GraphPad Prism 6 (GraphPad Software, CA, USA). Statistical significance was determined as a *P* value of less than 0.05.

## Results

### Participants

In total, 2,059 (1,097 ATB and 962 LTBI) and another 883 (372 ATB and 511 LTBI) participants were recruited in Qiaokou and Caidian cohorts, respectively (**Table 1**). Their demographic, clinical, and laboratory information was summarized in **Table 1**. There was no significant difference in age or sex distribution between individuals with ATB and LTBI in two cohorts. About 63% of subjects were male, and the mean age was around 50 years (**Table 1**).

**Table 1:** Demographic and clinical characteristics of study participants.

Variables	Qiaokou (training) cohort		P*	Caidian (validation) cohort		P*	*P* ^†^
	ATB (n = 1,097)	LTBI (n = 962)		ATB (n = 372)	LTBI (n = 511)		
Sex, male, %	63.26%	60.29%	0.173	65.32%	61.64%	0.289	0.507
Age, years	50.55 ± 16.80	51.84 ± 14.16	0.102	51.50 ± 17.04	52.10 ± 14.11	0.975	0.218
Presence of BCG scar	46.95%	39.92%	<0.001	45.70%	36.99%	<0.01	0.133
TB history	22.97%	0.00%	<0.001	23.12%	0.00%	<0.001	0.058
Underlying condition or illness							
HIV infection	0.36%	0.00%	0.128	0.27%	0.00%	0.421	1
Diabetes mellitus	7.66%	5.93%	0.121	9.95%	7.05%	0.122	0.174
End-stage renal disease	3.74%	2.49%	0.108	4.84%	2.74%	0.099	0.516
Liver cirrhosis	1.09%	0.73%	0.386	2.15%	1.17%	0.251	0.118
Hematological malignancy	2.83%	2.18%	0.354	3.23%	1.57%	0.102	0.675
Solid tumor	11.30%	9.04%	0.092	9.95%	7.24%	0.152	0.117
Organ transplantation	2.92%	1.77%	0.088	3.49%	1.76%	0.103	0.856
Other bacterial infection^‡^	3.19%	0.00%	<0.001	4.03%	0.00%	<0.001	0.998
Rheumatic immune diseases	4.10%	2.91%	0.145	3.76%	3.13%	0.609	0.841
Positive mycobacterial culture	86.96%	N/A	N/A	88.98%	N/A	N/A	N/A
Positive Xpert MTB/RIF	77.85%	N/A	N/A	79.57%	N/A	N/A	N/A

ATB, active tuberculosis; LTBI, latent tuberculosis infection; BCG, bacille Calmette-Guérin; TB, tuberculosis; N/A, not applicable. *Comparisons were performed between ATB and LTBI groups using chi-square test or Mann-Whitney U test. ^†^Comparisons were performed between Qiaokou and Caidian cohorts using chi-square test or Mann-Whitney U test. ^‡^Bacterial infection was diagnosed by microbiological evidences. Data were presented as means ± SD or percentages.

### Results of BRE and T-SPOT in Individuals ATB and LTBI in Qiaokou Cohort

**Figure 1:**
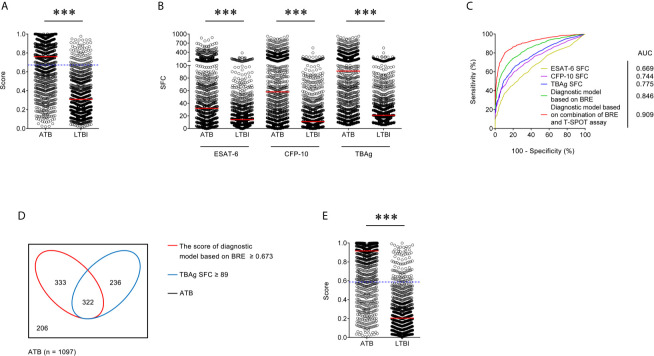
Establishment of diagnostic model based on combination of BRE and T-SPOT in Qiaokou cohort. **(A)** Scatter plots showing the score of diagnostic model based on BRE in ATB patients (n = 1,097) and LTBI individuals (n = 962) in Qiaokou cohort. Horizontal lines indicate the median. ***P < 0.001 (Mann-Whitney *U* test). Blue dotted lines indicate the cutoff value in distinguishing these two groups. **(B)** Scatter plots showing ESAT-6 SFC, CFP-10 SFC, and TBAg SFC in ATB patients (n = 1,097) and LTBI individuals (n = 962) in Qiaokou cohort. Horizontal lines indicate the median. ***P < 0.001 (Mann-Whitney *U* test). **(C)** ROC analysis showing the performance of ESAT-6 SFC, CFP-10 SFC, TBAg SFC, diagnostic model based on BRE, diagnostic based on combination of BRE and T-SPOT in distinguishing ATB from LTBI in Qiaokou cohort. **(D)** Venn diagrams showing the overlap of the diagnostic model based on BRE and TBAg SFC in ATB patients (n = 1,097) in Qiaokou cohort. **(E)** Scatter plots showing the score of diagnostic model based on combination of BRE and T-SPOT in ATB patients (n = 1,097) and LTBI individuals (n = 962) in Qiaokou cohort. Horizontal lines indicate the median. ***P < 0.001 (Mann-Whitney *U* test). Blue dotted lines indicate the cutoff values in distinguishing these two groups. ATB, active tuberculosis; LTBI, latent tuberculosis infection; ESAT-6, early secreted antigenic target 6; CFP-10, culture filtrate protein 10; TBAg, tuberculosis-specific antigens; SFC, spot-forming cells; AUC, area under the curve; BRE, blood routine examination.

All indicators in BRE differed significantly between individuals with ATB and LTBI in Qiaokou cohort. Specifically, WBC#, NEUT%, NEUT#, MONO%, MONO#, RDW-CV, RDW-SD, PLT#, and PCT in ATB were significantly higher than LTBI. In contrast, LYMPH%, LYMPH#, EO%, EO#, BASO%, BASO#, RBC#, HGB, HCT, MCV, MCH, MCHC, PDW, MPV, and PLCR in ATB were significantly lower than LTBI (**Table 2** and **Supplementary Figure 1**). For T-SPOT assay, ESAT-6 and CFP-10 SFC in ATB were significantly higher than LTBI (**Table 2** and **Figure 1**).

**Table 2:** The results of T-SPOT and blood routine examination of the study participants.

Variables	Qiaokou (training) cohort		P*	Caidian (validation) cohort		P*	*P* ^†^
	ATB (n = 1,097)	LTBI (n = 962)		ATB (n = 372)	LTBI (n = 511)		
ESAT-6 SFC	32 (11–93)	14 (7–31)	<0.001	30 (12–98)	12 (6–27)	<0.001	0.027
CFP-10 SFC	58 (14–167)	11 (4–30)	<0.001	54 (15–159)	10 (4–25)	<0.001	<0.001
WBC# (×10^9^/L)	6.47 (5.12–8.63)	5.94 (5.02–7.05)	<0.001	6.44 (5.26–8.31)	5.95 (5.08–7.16)	<0.001	0.553
NEUT% (%)	69.2 (61.8–76.4)	58.5 (53.2–64.3)	<0.001	69.2 (62.5–75.7)	59.6 (53.7–64.6)	<0.001	0.073
NEUT# (×10^9^/L)	4.46 (3.25–6.26)	3.46 (2.74–4.33)	<0.001	4.48 (3.32–6.13)	3.49 (2.81–4.47)	<0.001	0.306
LYMPH% (%)	20.1 (13.6–26.5)	30.3 (25.0–36.1)	<0.001	19.5 (13.8–25.8)	29.9 (25.2–34.8)	<0.001	0.078
LYMPH# (×10^9^/L)	1.24 (0.92–1.67)	1.79 (1.48–2.14)	<0.001	1.23 (0.91–1.62)	1.74 (1.49–2.07)	<0.001	0.129
MONO% (%)	8.1 (6.3–10.0)	7.4 (6.3–8.8)	<0.001	8.3 (6.4–10.4)	7.7 (6.4–9.0)	<0.001	0.329
MONO# (×10^9^/L)	0.51 (0.38–0.70)	0.45 (0.36–0.55)	<0.001	0.53 (0.39–0.69)	0.45 (0.37–0.55)	<0.001	0.853
EO% (%)	1.2 (0.5–2.4)	2.1 (1.2–3.3)	<0.001	1.2 (0.4–2.3)	1.9 (1.1–3.1)	<0.001	0.736
EO# (×10^9^/L)	0.07 (0.03–0.15)	0.12 (0.07–0.20)	<0.001	0.08 (0.03–0.15)	0.12 (0.06–0.19)	<0.001	0.661
BASO% (%)	0.3 (0.2–0.5)	0.4 (0.2–0.6)	<0.001	0.3 (0.2–0.5)	0.4 (0.2–0.6)	<0.001	0.098
BASO# (×10^9^/L)	0.02 (0.01–0.03)	0.02 (0.01–0.04)	<0.001	0.02 (0.01–0.03)	0.02 (0.02–0.03)	<0.001	0.097
RBC# (×10^12^/L)	4.30 (3.89–4.67)	4.53 (4.18–4.92)	<0.001	4.26 (3.97–4.67)	4.54 (4.18–4.91)	<0.001	0.097
HGB (g/L)	125 (113–137)	137 (126–150)	<0.001	126 (113–138)	137 (126–150)	<0.001	0.01
HCT (%)	37.8 (34.2–41.2)	40.9 (37.7–44.4)	<0.001	37.9 (34.4–41.2)	41.2 (37.7–44.5)	<0.001	0.019
MCV (fl)	88.2 (84.8–91.8)	90.4 (87.8–93.2)	<0.001	87.9 (84.3–91.6)	90.5 (87.7–92.8)	<0.001	0.695
MCH (pg)	29.3 (27.9–30.6)	30.3 (29.4–31.3)	<0.001	29.2 (27.9–30.6)	30.3 (29.3–31.3)	<0.001	0.335
MCHC (g/L)	332 (321–340)	335 (328–341)	<0.001	331 (323–340)	335 (329–342)	<0.001	0.16
RDW-CV	13.4 (12.7–14.2)	12.9 (12.4–13.3)	<0.001	13.3 (12.6–14.2)	12.8 (12.3–13.3)	<0.001	0.007
RDW-SD (fl)	42.9 (40.3–45.6)	42.4 (40.4–44.2)	<0.001	42.7 (40.1–46.2)	42.1 (40.5–43.9)	0.010	0.158
PLT# (×10^9^/L)	235 (177–296)	215 (180–254)	<0.001	242 (187–311)	216 (184–257)	<0.001	0.393
PDW (fl)	11.8 (10.3–14.0)	12.5 (11.2–14.4)	<0.001	11.9 (10.3–13.5)	12.5 (11.2–14.3)	<0.001	0.752
MPV (fl)	10.1 (9.4–11.1)	10.5 (9.9–11.4)	<0.001	10.1 (9.4–10.9)	10.5 (9.8–11.3)	<0.001	0.776
PLCR (%)	26.6 (20.7–34.2)	29.6 (24.3–36.6)	<0.001	26.7 (20.4–32.6)	29.2 (24.0–36.0)	<0.001	0.793
PCT (%)	0.24 (0.19–0.29)	0.23 (0.19–0.26)	0.002	0.24 (0.20–0.30)	0.23 (0.19–0.27)	<0.001	0.459

ATB, active tuberculosis; LTBI, latent tuberculosis infection; TB, tuberculosis; ESAT-6, early secreted antigenic target 6; CFP-10, culture filtrate protein 10; SFC, spot-forming cells; WBC#, white blood cell count; NEUT%, neutrophil percentage; NEUT#, neutrophil count; LYMPH%, lymphocyte percentage; LYMPH#, lymphocyte count; MONO%, monocyte percentage; MONO#, monocyte count; EO%, eosinophil percentage; EO#, eosinophil count; BASO%, basophil percentage; BASO#, basophil count; RBC#, red blood cell count; HGB, hemoglobin; HCT, hematocrit; MCV, mean corpuscular volume; MCH, mean corpuscular hemoglobin; MCHC, mean corpuscular hemoglobin concentration; RDW-CV, coefficient variation of  red blood cell volume distribution width; RDW-SD, standard deviation in red cell distribution width; PLT#, platelet count; PDW, platelet distribution width; MPV, mean platelet volume; PLCR, platelet larger cell ratio; PCT, thrombocytocrit. *Comparisons were performed between ATB and LTBI groups using chi-square test or Mann-Whitney U test. ^†^Comparisons were performed between Qiaokou and Caidian cohorts using chi-square test or Mann-Whitney U test. Data were presented as medians (25th–75th percentages).

### Diagnostic Model Base on BRE for Differentiating ATB From LTBI in Qiaokou Cohort

To establish a diagnostic model based on indicators in BRE for distinguishing ATB from LTBI, all variables with statistical significance were used for multivariable logistic regression analysis. A diagnostic model was built as the following: P = 1/[1 + e^−(−0.59 + 0.377 × NEUT# − 1.579 × LYMPH# + 1.78 × MONO# − 1.929 × EO# − 0.028 × HGB + 0.327 × RDW-CV + 0.002 × PLT#)^] P, predictive value; e, natural logarithm. ROC analysis showed that the AUC of the diagnostic model was 0.846 (95% CI, 0.829 to 0.862) (**Figures 1A, C**). When the cutoff value was set at 0.673, the sensitivity and specificity were 59.71 and 91.58%, respectively (**Table 3**).

**Table 3:** The performance of various methods for distinguishing between ATB and LTBI in Qiaokou cohort.

Variables	Cutoff value	AUC (95% CI)	Sensitivity (95% CI)	Specificity (95% CI)	PPV (95% CI)	NPV (95% CI)	PLR (95% CI)	NLR (95% CI)	Accuracy
ESAT-6 SFC	61	0.669 (0.646–0.692)	35.73% (32.95–38.61%)	90.23% (88.19–91.95%)	80.66% (76.91–83.92%)	55.18% (52.71–57.62%)	3.66 (2.97–4.5)	0.71 (0.68–0.75)	61.19%
CFP-10 SFC	76	0.744 (0.723–0.765)	44.03% (41.12–46.98%)	90.85% (88.86–92.52%)	84.59% (81.40–87.32%)	58.74% (56.22–61.21%)	4.81 (3.90–5.94)	0.62 (0.58–0.65)	65.91%
TBAg SFC	89	0.775 (0.755–0.795)	50.77% (47.82–53.73%)	90.12% (88.08–91.85%)	85.43% (82.51–87.93%)	61.62% (59.05–64.13%)	5.14 (4.21–6.28)	0.55 (0.51–0.58)	69.16%
Diagnostic model based on BRE	0.673	0.846 (0.829–0.862)	59.71% (56.78–62.57%)	91.58% (89.66–93.17%)	88.99% (86.53–91.06%)	66.59% (64.00–69.08%)	7.09 (5.73–8.78)	0.44 (0.41–0.47)	74.60%
Diagnostic model based on combination of BRE and T-SPOT assay	0.587	0.909 (0.897–0.922)	78.03% (75.49–80.38%)	90.23% (88.19–91.95%)	90.11% (88.04–91.85%)	78.27% (75.75–80.60%)	7.99 (6.57–9.70)	0.24 (0.22–0.27)	83.73%

ATB, active tuberculosis; LTBI, latent tuberculosis infection; ESAT-6, early secreted antigenic target 6; CFP-10, culture filtrate protein 10; TBAg, tuberculosis-specific antigens; SFC, spot-forming cells; BRE, blood routine examination; AUC, area under the curve; PPV, positive predictive value; NPV, negative predictive value; PLR, positive likelihood ratio; NLR, negative likelihood ratio; CI, confidence interval.

### The Performance of T-SPOT for Distinguishing Between ATB and LTBI in Qiaokou Cohort

In Qiaokou cohort, ROC analysis showed that the AUC of ESAT-6 SFC in distinguishing ATB from LTBI was 0.669 (95% CI 0.646 to 0.692), with a sensitivity of 35.73% and a specificity of 90.23% at the cutoff value of 61. Meanwhile, The AUC of the ROC curve for CFP-10 SFC was 0.744 (95% CI 0.723 to 0.765), with a sensitivity of 44.03% and a specificity of 90.85% when a threshold value of 76 was used. Moreover, when using TBAg SFC as an indicator, the sensitivity and specificity in distinguishing these two conditions were 50.77 and 90.12% respectively with a threshold of 89 (**Table 3** **** and **Figures 1B, C**).

### Diagnostic Model Based on Combination of BRE and T-SPOT for Discriminating ATB and LTBI in Qiaokou Cohort

Although either BRE or T-SPOT showed potential value in ATB and LTBI discrimination, both of their sensitivities were relatively low. However, the overlap between TBAg SFC and diagnostic model based on BRE showed that combination of these two methods could improve the diagnostic effect (**Figure 1D**). Thus, a new diagnostic model was obtained by logistic regression analysis as the following: P = 1/[1 + e^−(−1.436 + 0.01 × ESAT-6 SFC + 0.011 × CFP-10 SFC + 0.403 × NEUT# − 1.835 × LYMPH# + 1.355 × MONO# − 2.225 × EO# − 0.027 × HGB + 0.31 × RDW-CV + 0.004 × PLT#)^] P, predictive value; e, natural logarithm. ROC analysis showed the AUC of the model to differentiate ATB from LTBI was 0.909 (95% CI, 0.889 to 0.930), with a sensitivity of 78.85% and a specificity of 90.23% when using 0.587 as the cutoff value (**Table 3** **** and **Figures 1C, E**).

### Validation of Diagnostic Model in Caidian Cohort

**Figure 2:**
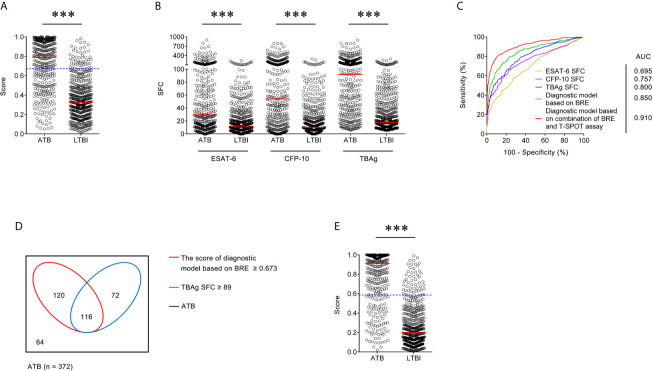
Validation of diagnostic model based on combination of BRE and T-SPOT in Caidian cohort. **(A)** Scatter plots showing the score of diagnostic model based on BRE in ATB patients (n = 372) and LTBI individuals (n = 511) in Caidian cohort. Horizontal lines indicate the median. ***P < 0.001 (Mann-Whitney *U* test). Blue dotted lines indicate the cutoff value in distinguishing these two groups. **(B)** Scatter plots showing ESAT-6 SFC, CFP-10 SFC, and TBAg SFC in ATB patients (n = 372) and LTBI individuals (n = 511) in Caidian cohort. Horizontal lines indicate the median. ***P < 0.001 (Mann-Whitney *U* test). **(C)** ROC analysis showing the performance of ESAT-6 SFC, CFP-10 SFC, TBAg SFC, diagnostic model based on BRE, diagnostic based on combination of BRE and T-SPOT in distinguishing ATB from LTBI in Caidian cohort. **(D)** Venn diagrams showing the overlap of the diagnostic model based on BRE and TBAg SFC in ATB patients (n = 372) in Caidian cohort. **(E)** Scatter plots showing the score of diagnostic model based on combination of BRE and T-SPOT in ATB patients (n = 372) and LTBI individuals (n = 511) in Caidian cohort. Horizontal lines indicate the median. ***P < 0.001 (Mann-Whitney *U* test). Blue dotted lines indicate the cutoff values in distinguishing these two groups. ATB, active tuberculosis; LTBI, latent tuberculosis infection; ESAT-6, early secreted antigenic target 6; CFP-10, culture filtrate protein 10; TBAg, tuberculosis-specific antigens; SFC, spot-forming cells; AUC, area under the curve; BRE, blood routine examination.

There was significant difference in all indicators in BRE between individuals with ATB and LTBI in Caidian cohort (**Table 2** and **Supplementary Figure 2**). Similar performance was observed in Caidian cohort. Validation of the diagnostic model based on BRE produced an AUC of 0.850 (95% CI, 0.823 to 0.876) with 63.44% sensitivity and 90.61% specificity (**Table 4** **** and **Figure 2A, C**). If using 89 that obtained from the training cohort as the cutoff value of TBAg SFC, the sensitivity and specificity were 50.54 and 92.37% in differentiating ATB from LTBI, respectively (**Table 4** **** and **Figures 2B, C**). The diagnostic model based on combination of BRE and T-SPOT also performed well in the validation cohort: 0.910 (95% CI, 0.889 to 0.930) AUC, 78.23% sensitivity, and 90.02% specificity (**Table 4** **** and **Figures 2C–E**).

**Table 4:** The performance of various methods for distinguishing between ATB and LTBI in Caidian cohort.

Variables	Cutoff	AUC (95% CI)	Sensitivity (95% CI)	Specificity (95% CI)	PPV (95% CI)	NPV (95% CI)	PLR (95% CI)	NLR (95% CI)	Accuracy
ESAT-6 SFC	61	0.695 (0.660–0.731)	36.02% (31.31–41.02%)	90.41% (87.55–92.67%)	73.22% (66.38–79.11%)	66.00% (62.41–69.41%)	3.76 (2.79–5.06)	0.71 (0.65–0.77)	67.50%
CFP-10 SFC	76	0.757 (0.723–0.790)	42.20% (37.29–47.28%)	92.95% (90.40–94.87%)	81.35% (75.26–86.21%)	68.84% (65.29–72.18%)	5.99 (4.28–8.39)	0.62 (0.57–0.68)	71.57%
TBAg SFC	89	0.800 (0.770–0.830)	50.54% (45.48–55.59%)	92.37% (89.74–94.37%)	82.82% (77.38–87.17%)	71.95% (68.39–75.25%)	6.62 (4.82–9.10)	0.54 (0.48–0.60)	74.75%
Diagnostic model based on BRE	0.673	0.850 (0.823–0.876)	63.44% (58.43–68.17%)	90.61% (87.77–92.84%)	83.10% (78.31–87.01%)	77.30% (73.77–80.47%)	6.75 (5.10–8.94)	0.40 (0.35–0.46)	79.16%
Diagnostic model based on combination of BRE and T-SPOT assay	0.587	0.910 (0.889–0.930)	78.23% (73.75–82.12%)	90.02% (87.11–92.33%)	85.09% (80.92–88.47%)	85.03% (81.77–87.79%)	7.84 (6.01–10.22)	0.24 (0.20–0.29)	85.05%

ATB, active tuberculosis; LTBI, latent tuberculosis infection; ESAT-6, early secreted antigenic target 6; CFP-10, culture filtrate protein 10; TBAg, tuberculosis-specific antigens; SFC, spot-forming cells; BRE, blood routine examination; AUC, area under the curve; PPV, positive predictive value; NPV, negative predictive value; PLR, positive likelihood ratio; NLR, negative likelihood ratio; CI, confidence interval.

### The Performance of Diagnostic Model for Differentiating ATB From LTBI With Lung Lesions

**Figure 3:**
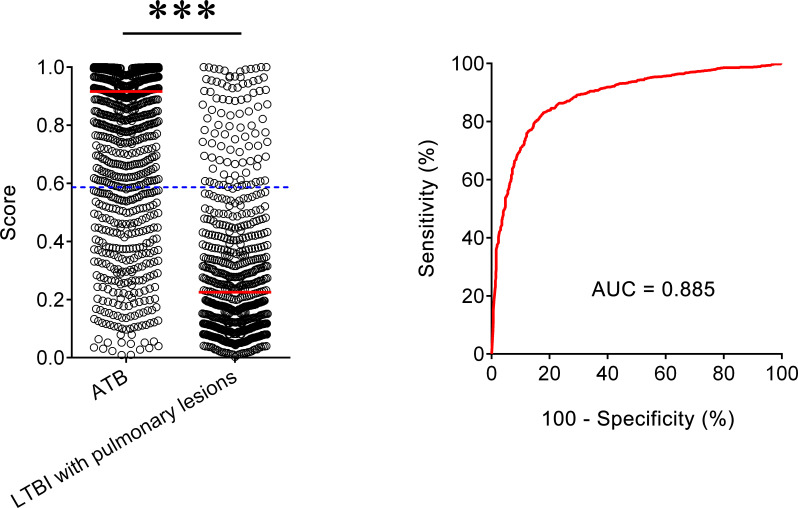
The performance of established model for discriminating ATB from LTBI with pulmonary lesions. Scatter plots showing the score of the diagnostic model in ATB patients (n = 1,097) and LTBI individuals with pulmonary lesions (n = 671). Horizontal lines indicate the median. ***P < 0.001 (Mann-Whitney *U* test). Blue dotted lines indicate the cutoff value in distinguishing these two groups. ROC analysis showing the performance of the diagnostic model in distinguishing ATB from LTBI with pulmonary lesions. ATB, active tuberculosis; LTBI, latent tuberculosis infection; AUC, area under the curve.

We also included another LTBI group with lung cancer to assess the established diagnostic model for differentiating ATB from LTBI with lung lesions. It was observed that the AUC of our model to distinguish ATB form this LTBI group was 0.885 (95% CI, 0.869–0.901) (**Figure 3**). When 0.587 was used as the threshold, the sensitivity and specificity of the model was 78.03% (95% CI, 75.49–80.38%) and 85.69% (95% CI, 82.84–88.14%), respectively. These data indicated that our diagnostic model was also useful for differentiating ATB from LTBI with pulmonary lesions.

## Discussion

TB remains an important infectious disease worldwide (Haas et al., 2016; Furin et al., 2019). To achieve TB elimination strategy, rapid, inexpensive, and accurate methods for differentiating between ATB and LTBI are urgently required, especially in high-endemic countries such as China (World Health Organization, 2016; Gao et al., 2017; Xin et al., 2019). The conventional pathogen-detecting methods have limitations in clinical application (MacLean et al., 2019). And on the other hand, although reports on new candidate markers are numerous over the last decade, there is rarely one marker which is suitable for clinical use (Burel et al., 2018; Meier et al., 2018; Singhania et al., 2018; Walzl et al., 2018; Silveira-Mattos et al., 2019; Warsinske et al., 2019). Therefore, the improvement based on pre-existing methods is particularly important.

BRE is one of the most common tests performed in clinical laboratory. The test is not only used for the differential diagnosis of infectious diseases, but is also used for many aspects such as inflammatory state assessment and nutritional status assessment. BRE is so widely used that almost all inpatients will be prescribed this test. Because of its wide application in many fields, few people realize that this routine test could be used in the diagnosis of TB. A previous study has shown that the ratio of neutrophils to lymphocytes has some value in diagnosing TB (Yoon et al., 2013). In our study, we observed that the percentage of neutrophils and lymphocytes was significantly increased and decreased respectively in ATB patients, compared with LTBI individuals. However, although the ratio of neutrophils to lymphocytes shows significant difference in these two groups, the performance of this ratio in diagnosing ATB was limited. After all, the similar change in neutrophils and lymphocytes can be occurred in many other diseases. Unexpectedly, we found except for neutrophils and lymphocytes, many indicators of BRE (i.e., MONO#, EO#) were all significantly different between ATB and LTBI. Thus, the diagnostic model base on combination of these indicators in BRE is of potential value in differentiating ATB from LTBI.

The present study recruited approximately 3,000 participants to examine the feasibility and efficiency of measuring biomarkers in BRE and T-SPOT for differential diagnosis of ATB and LTBI. This is the basis for us to observe a statistical difference in BRE and T-SPOT between these two groups. However, although either BRE or T-SPOT showed significant difference between ATB and LTBI, using BRE or T-SPOT alone had limited diagnostic accuracy in distinguishing these two states. Interestingly, by the combination of these two tests, we successfully established a diagnostic model that showed good performance in distinguishing ATB from LTBI. The AUC and accuracy for the diagnostic model in both the training and validation cohorts were ~0.90 and 85%, respectively.

Given that T-SPOT assay detects Mtb-specific response of lymphocytes, it is understandable that the results of T-SPOT in ATB patients were higher than in LTBI individuals. However, we unexpectedly found that many indicators in BRE also showed significant difference between ATB and LTBI. In accordance with a previous study (Iliaz et al., 2014), we observed that the number of lymphocytes was decreased in ATB patients, which may be caused by that the host immunity, especially T cells and NK cells, was impaired in the pathogenesis of TB. Moreover, malnutrition is one of the most important risk factors for the development of ATB in Mtb-infected individuals (Cegielski and McMurray, 2004). Thus, it is reasonable that HGB was significant decreased in ATB patients compared with LTBI. But we don’t know why many other indicators such as EO# and PLT# also have significant difference between ATB and LTBI. A further study is needed to determine the role of coagulation system in the pathogenesis of TB.

Another interesting question is why T-SPOT assay and BRE have complementary effects on TB diagnosis. We speculate that T-SPOT and BRE exhibit different performance in Mtb-infected individuals with different immune status. Previous studies have showed that T-SPOT results were obviously decreased in immunocompromised ATB patients (Bosco et al., 2017). Therefore, it is very difficult to distinguish ATB from LTBI in this condition because most times those low T-SPOT results are attributed to LTBI. However, many indicators in BRE including LYMPH# and HGB were decreased in immunocompromised patients. Thus, the performance of BRE in distinguishing ATB from LTBI may be better in immunocompromised patients than immunocompetent ones. In contrast, it is reasonable that the performance of T-SPOT is better than BRE in distinguishing ATB from LTBI in immunocompetent patients.

Two limitations of this study should be noted. First, although the number of participants in this study was relatively large, all of them were recruited from one city in China, which may not represent the status of patients globally. Future investigations are now required to validate and optimize this model in larger cohorts recruited from other clinical centers worldwide. Second, since the subjects enrolled in this study were all 17 years of age and older, the performance of the diagnostic model in individuals under 17 years of age was unknown.

In conclusion, this is the first study of using BRE and T-SPOT to establish diagnostic model for discriminating ATB from LTBI in a large number of participants and this model might serve as a simple, innovative, and attractive strategy for TB diagnosis, particularly in TB-endemic areas.

## Data Availability Statement

The original contributions presented in the study are included in the article/**Supplementary Material**. Further inquiries can be directed to the corresponding authors.

## Ethics Statement

The studies involving human participants were reviewed and approved by the Ethics Committee of Tongji Hospital, Tongji Medical College, Huazhong University of Science and Technology, Wuhan, China. The patients/participants provided their written informed consent to participate in this study.

## Author Contributions

YL, FW, and ZS conceived the study. YL, GT, XY, QL, LM, HS, and YX recruited and assessed the patients and preprocessed the study specimens. YL, SW, RO, and HH were responsible for statistical analysis. YL and FW interpreted the data and wrote the manuscript. All authors contributed to the article and approved the submitted version.

## Funding

This work was supported by research grants from the Graduate Innovation Fund of Huazhong University of Science and Technology (grant number 2021yjsCXCY088), the National Mega Project on Major Infectious Disease Prevention (2017ZX10103005-007), and the National Natural Science Foundation of China (81401639).

## Conflict of Interest

The authors declare that the research was conducted in the absence of any commercial or financial relationships that could be construed as a potential conflict of interest.

## Supplementary Material

Supplementary Figure 1 The results of BRE in ATB patients (n = 1,097) and LTBI individuals (n = 962) in Qiaokou cohort. ***P* < 0.01, ****P* < 0.001 (Mann-Whitney *U* test). WBC#, white blood cell count; NEUT%, neutrophil percentage; NEUT#, neutrophil count; LYMPH%, lymphocyte percentage; LYMPH#, lymphocyte count; MONO%, monocyte percentage; MONO#, monocyte count; EO%, eosinophil percentage; EO#, eosinophil count; BASO%, basophil percentage; BASO#, basophil count; RBC#, red blood cell count; HGB, hemoglobin; HCT, hematocrit; MCV, mean corpuscular volume; MCH, mean corpuscular hemoglobin; MCHC, mean corpuscular hemoglobin concentration; RDW-CV, coefficient variation of  red blood cell volume distribution width; RDW-SD, standard deviation in red cell distribution width; PLT#, platelet count; PDW, platelet distribution width; MPV, mean platelet volume; PLCR, platelet larger cell ratio; PCT, thrombocytocrit. ATB, active tuberculosis; LTBI, latent tuberculosis infection; BRE, blood routine examination.

Supplementary Figure 2 The results of BRE in ATB patients (n = 372) and LTBI individuals (n = 511) in Caidian cohort. ***P* < 0.01, ****P* < 0.001 (Mann-Whitney *U* test). WBC#, white blood cell count; NEUT%, neutrophil percentage; NEUT#, neutrophil count; LYMPH%, lymphocyte percentage; LYMPH#, lymphocyte count; MONO%, monocyte percentage; MONO#, monocyte count; EO%, eosinophil percentage; EO#, eosinophil count; BASO%, basophil percentage; BASO#, basophil count; RBC#, red blood cell count; HGB, hemoglobin; HCT, hematocrit; MCV, mean corpuscular volume; MCH, mean corpuscular hemoglobin; MCHC, mean corpuscular hemoglobin concentration; RDW-CV, coefficient variation of  red blood cell volume distribution width; RDW-SD, standard deviation in red cell distribution width; PLT#, platelet count; PDW, platelet distribution width; MPV, mean platelet volume; PLCR, platelet larger cell ratio; PCT, thrombocytocrit. ATB, active tuberculosis; LTBI, latent tuberculosis infection; BRE, blood routine examination.

## References

[B1] Adekambi T. Ibegbu C. C. Cagle S. Kalokhe A. S. Wang Y. F. Hu Y.. (2015). Biomarkers on Patient T Cells Diagnose Active Tuberculosis and Monitor Treatment Response. J. Clin. Invest. 125 (5), 1827–1838. 10.1172/JCI77990 25822019 PMC4598074

[B2] Blumberg H. M. Ernst J. D. (2016). The Challenge of Latent Tb Infection. JAMA 316 (9), 931–933. 10.1001/jama.2016.11021 27599327 PMC5319563

[B3] Bosco M. J. Hou H. Mao L. Wu X. Ramroop K. D. Lu Y.. (2017). The Performance of the TBAg/PHA Ratio in the Diagnosis of Active TB Disease in Immunocompromised Patients. Int. J. Infect. Dis. 59, 55–60. 10.1016/j.ijid.2017.03.025 28392318

[B4] Burel J. G. Lindestam Arlehamn C. S. Khan N. Seumois G. Greenbaum J. A. Taplitz R.. (2018). Transcriptomic Analysis of CD4(+) T Cells Reveals Novel Immune Signatures of Latent Tuberculosis. J. Immunol. 200 (9), 3283–3290. 10.4049/jimmunol.1800118 29602771 PMC5991485

[B5] Cegielski J. P. McMurray D. N. (2004). The Relationship Between Malnutrition and Tuberculosis: Evidence From Studies in Humans and Experimental Animals. Int. J. Tuberc. Lung Dis. 8 (3), 286–298. 15139466

[B6] Chaisson R. Penn-Nicholson A. Hraha T. Thompson E. G. Sterling D. Mbandi S. K.. (2019). Discovery and Validation of a Prognostic Proteomic Signature for Tuberculosis Progression: A Prospective Cohort Study. PloS Med. 16 (4), e1002781. 10.1371/journal.pmed.1002781 30990820 PMC6467365

[B7] Chakravorty S. Simmons A. M. Rowneki M. Parmar H. Cao Y. Ryan J.. (2017). The New Xpert MTB/RIF Ultra: Improving Detection of Mycobacterium Tuberculosis and Resistance to Rifampin in an Assay Suitable for Point-of-Care Testing. MBio 8 (4), e00812–17. 10.1128/mBio.00812-17 28851844 PMC5574709

[B8] Cohen A. Mathiasen V. D. Schön T and Wejse C. (2019). The Global Prevalence of Latent Tuberculosis: A Systematic Review and Meta-Analysis. Eur. Respir. J. 54 (3), 1900655. 10.1183/13993003.00655-2019 31221810

[B9] Dai Y. Shan W. Yang Q. Guo J. Zhai R. Tang X.. (2019). Biomarkers of Iron Metabolism Facilitate Clinical Diagnosis in M Ycobacterium Tuberculosis Infection. Thorax 74 (12), 1161–1167. 10.1136/thoraxjnl-2018-212557 31611342 PMC6902069

[B10] Dorman S. E. Schumacher S. G. Alland D. Nabeta P. Armstrong D. T. King B.. (2018). Xpert MTB/RIF Ultra for Detection of Mycobacterium Tuberculosis and Rifampicin Resistance: A Prospective Multicentre Diagnostic Accuracy Study. Lancet Infect. Dis. 18 (1), 76–84. 10.1016/S1473-3099(17)30691-6 29198911 PMC6168783

[B11] Furin J. Cox H. Pai M. (2019). Tuberculosis. Lancet 393 (10181), 1642–1656. 10.1016/S0140-6736(19)30308-3 30904262

[B12] Gao L. Li X. Liu J. Wang X. Lu W. Bai L.. (2017). Incidence of Active Tuberculosis in Individuals With Latent Tuberculosis Infection in Rural China: Follow-Up Results of a Population-Based, Multicentre, Prospective Cohort Study. Lancet Infect. Dis. 17 (10), 1053–1061. 10.1016/S1473-3099(17)30402-4 28716677

[B13] Haas C. T. Roe J. K. Pollara G. Mehta M. Noursadeghi M. (2016). Diagnostic ‘Omics’ for Active Tuberculosis. BMC Med. 14, 37. 10.1186/s12916-016-0583-9 27005907 PMC4804573

[B14] Horne D. J. Kohli M. Zifodya J. S. Schiller I. Dendukuri N. Tollefson D.. (2019). Xpert MTB/RIF and Xpert Mtb/Rif Ultra for Pulmonary Tuberculosis and Rifampicin Resistance in Adults. Cochrane Database Syst. Rev. 6, CD009593. 10.1002/14651858.CD009593.pub4 31173647 PMC6555588

[B15] Iliaz S. Iliaz R. Ortakoylu G. Bahadir A. Bagci B. A. Caglar E. (2014). Value of Neutrophil/Lymphocyte Ratio in the Differential Diagnosis of Sarcoidosis and Tuberculosis. Ann. Thorac. Med. 9 (4), 232–235. 10.4103/1817-1737.140135 25276243 PMC4166071

[B16] MacLean E. Broger T. Yerliyaka S. Fernandez-Carballo B. L. Pai M. Denkinger C. M. (2019). A Systematic Review of Biomarkers to Detect Active Tuberculosis. Nat. Microbiol. 4 (5), 748–758. 10.1038/s41564-019-0380-2 30804546

[B17] Meier N. R. Jacobsen M. Ottenhoff T. H. M. Ritz N. (2018). A Systematic Review on Novel Mycobacterium Tuberculosis Antigens and Their Discriminatory Potential for the Diagnosis of Latent and Active Tuberculosis. Front. Immunol. 9, 2476. 10.3389/fimmu.2018.02476 30473692 PMC6237970

[B18] Musvosvi M. Duffy D. Filander E. Africa H. Mabwe S. Jaxa L.. (2018). T-Cell Biomarkers for Diagnosis of Tuberculosis: Candidate Evaluation by a Simple Whole Blood Assay for Clinical Translation. Eur. Respir. J. 51 (3), 1800153. 10.1183/13993003.00153-2018 29567725

[B19] Roe J. Venturini C. Gupta R. K. Gurry C. Chain B. M. Sun Y.. (2019). Blood Transcriptomic Stratification of Short-Term Risk in Contacts of Tuberculosis. Clin. Infect. Dis. 70 (5), 731–737. 10.1093/cid/ciz252 PMC7617764 30919880

[B20] Roy Chowdhury R. Vallania F. Yang Q. Lopez Angel C. J. Darboe F. Penn-Nicholson A.. (2018). A Multi-Cohort Study of the Immune Factors Associated With M. Tuberculosis Infection Outcomes. Nature 560 (7720), 644–648. 10.1038/s41586-018-0439-x 30135583 PMC6414221

[B21] Silveira-Mattos P. S. Barreto-Duarte B. Vasconcelos B. Fukutani K. F. Vinhaes C. L. Oliveira-de-Souza D.. (2019). Differential Expression of Activation Markers by Mycobacterium Tuberculosis-Specific CD4+ T-Cell Distinguishes Extrapulmonary From Pulmonary Tuberculosis and Latent Infection. Clin. Infect. Dis. 71 (8), 1905–1911. 10.1093/cid/ciz1070 PMC8463092 31665254

[B22] Singhania A. Verma R. Graham C. M. Lee J. Tran T. Richardson M.. (2018). A Modular Transcriptional Signature Identifies Phenotypic Heterogeneity of Human Tuberculosis Infection. Nat. Commun. 9 (1), 2308. 10.1038/s41467-018-04579-w 29921861 PMC6008327

[B23] Singhania A. Wilkinson R. J. Rodrigue M. Haldar P. O’Garra A. (2018). The Value of Transcriptomics in Advancing Knowledge of the Immune Response and Diagnosis in Tuberculosis. Nat. Immunol. 19 (11), 1159–1168. 10.1038/s41590-018-0225-9 30333612 PMC6554194

[B24] Suliman S. Thompson E. Sutherland J. Weiner Rd J. Ota M. O. C. Shankar S.. (2018). Four-Gene Pan-African Blood Signature Predicts Progression to Tuberculosis. Am. J. Respir. Crit. Care Med. 197 (9), 1198–1208. 10.1164/rccm.201711-2340OC 29624071 PMC6019933

[B25] Turetz M. L. Ma K. C. (2016). Diagnosis and Management of Latent Tuberculosis. Curr. Opin. Infect. Dis. 29 (2), 205–211. 10.1097/QCO.0000000000000253 26836374

[B26] Walzl G. McNerney R. du Plessis N. Bates M. McHugh T. D. Chegou N. N.. (2018). Tuberculosis: Advances and Challenges in Development of New Diagnostics and Biomarkers. Lancet Infect. Dis. 18 (7), e199–e210. 10.1016/S1473-3099(18)30111-7 29580818

[B27] Warsinske H. Vashisht R. Khatri P. (2019). Host-Response-Based Gene Signatures for Tuberculosis Diagnosis: A Systematic Comparison of 16 Signatures. PloS Med. 16 (4), e1002786. 10.1371/journal.pmed.1002786 31013272 PMC6478271

[B28] Weiner J. 3rd Maertzdorf J. Sutherland J. S. Duffy F. J. Thompson E. Suliman S.. (2018). Metabolite Changes in Blood Predict the Onset of Tuberculosis. Nat. Commun. 9 (1), 5208. 10.1038/s41467-018-07635-7 30523338 PMC6283869

[B29] Won E. J. Choi J. H. Cho Y. N. Jin H. M. Kee H. J. Park Y. W.. (2017). Biomarkers for Discrimination Between Latent Tuberculosis Infection and Active Tuberculosis Disease. J. Infect. 74 (3), 281–293. 10.1016/j.jinf.2016.11.010 27871809

[B30] World Health Organization (2015). Implementing Tuberculosis Diagnostics. Policy Framework.

[B31] World Health Organization (2016). The End TB Strategy 2015 (Geneva: World Health Organization).

[B32] World Health Organization (2019) Global Tuberculosis Report 2019. Available at: https://www.who.int/tb/publications/global_report/en/.

[B33] Xin H. Zhang H. Yang S. Liu J. Lu W. Bai L.. (2019). 5-Year Follow-Up of Active Tuberculosis Development From Latent Infection in Rural China. Clin. Infect. Dis. 70 (5), 947–950. 10.1093/cid/ciz581 31253988

[B34] Yoon N. B. Son C. Um S. J. (2013). Role of the Neutrophil-Lymphocyte Count Ratio in the Differential Diagnosis Between Pulmonary Tuberculosis and Bacterial Community-Acquired Pneumonia. Ann. Lab. Med. 33 (2), 105–110. 10.3343/alm.2013.33.2.105 23482854 PMC3589634

