# Supplementary figures and images for: Combination of Blood Routine Examination and T-SPOT.TB Assay for Distinguishing Between Active Tuberculosis and Latent Tuberculosis Infection
Source: Front Cell Infect Microbiol. 2021 Jun 29;11:575650. doi: 10.3389/fcimb.2021.575650 (PMC8279757; doi:10.3389/fcimb.2021.575650)

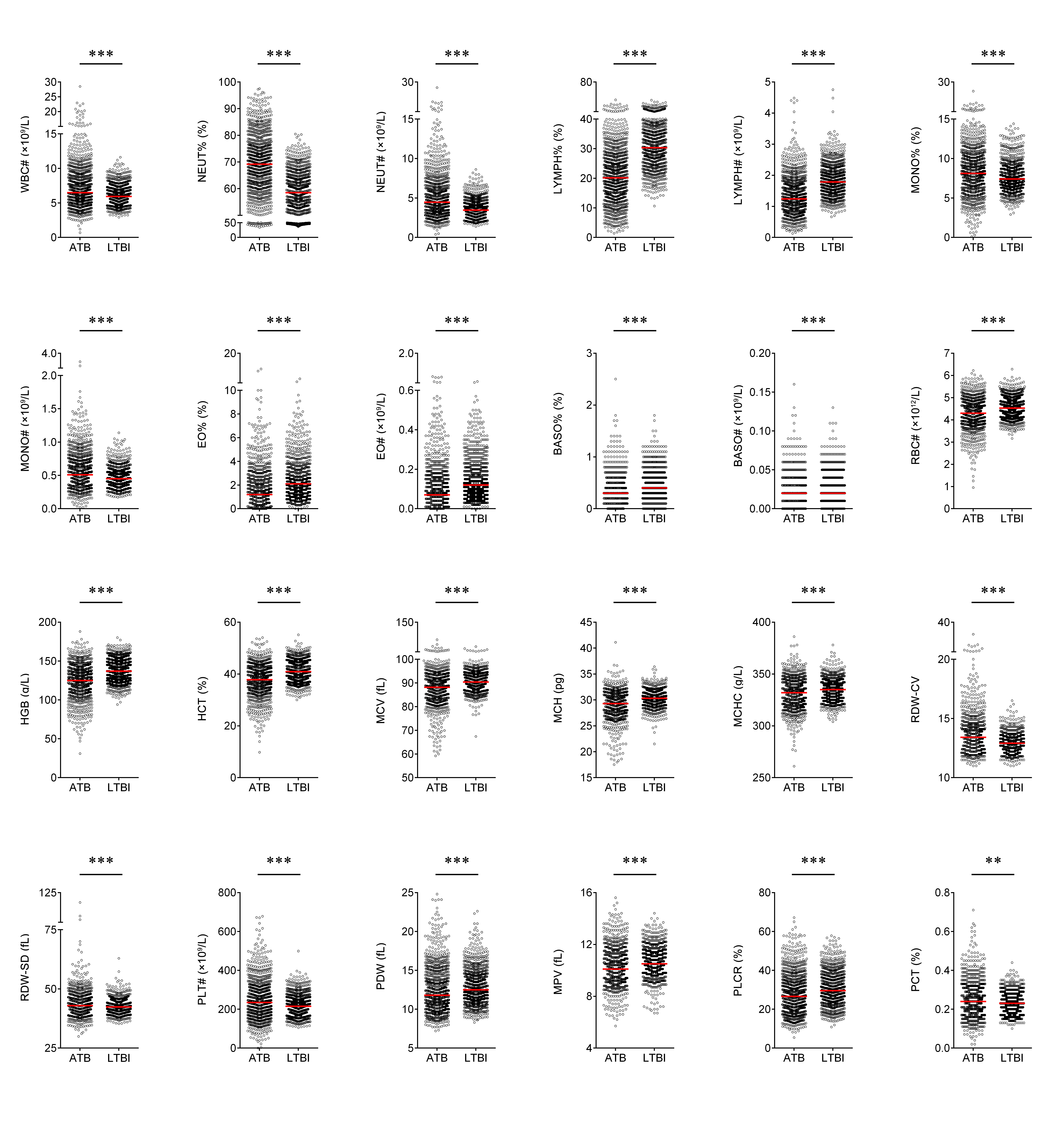

Supplement: Supplementary Figure 1 — The results of BRE in ATB patients (n = 1,097) and LTBI individuals (n = 962) in Qiaokou cohort. **P < 0.01, ***P < 0.001 (Mann-Whitney U test). WBC#, white blood cell count; NEUT%, neutrophil percentage; NEUT#, neutrophil count; LYMPH%, lymphocyte percentage; LYMPH#, lymphocyte count; MONO%, monocyte percentage; MONO#, monocyte count; EO%, eosinophil percentage; EO#, eosinophil count; BASO%, basophil percentage; BASO#, basophil count; RBC#, red blood cell count; HGB, hemoglobin; HCT, hematocrit; MCV, mean corpuscular volume; MCH, mean corpuscular hemoglobin; MCHC, mean corpuscular hemoglobin concentration; RDW-CV, coefficient variation of red blood cell volume distribution width; RDW-SD, standard deviation in red cell distribution width; PLT#, platelet count; PDW, platelet distribution width; MPV, mean platelet volume; PLCR, platelet larger cell ratio; PCT, thrombocytocrit. ATB, active tuberculosis; LTBI, latent tuberculosis infection; BRE, blood routine examination. [file Image_1.tif]

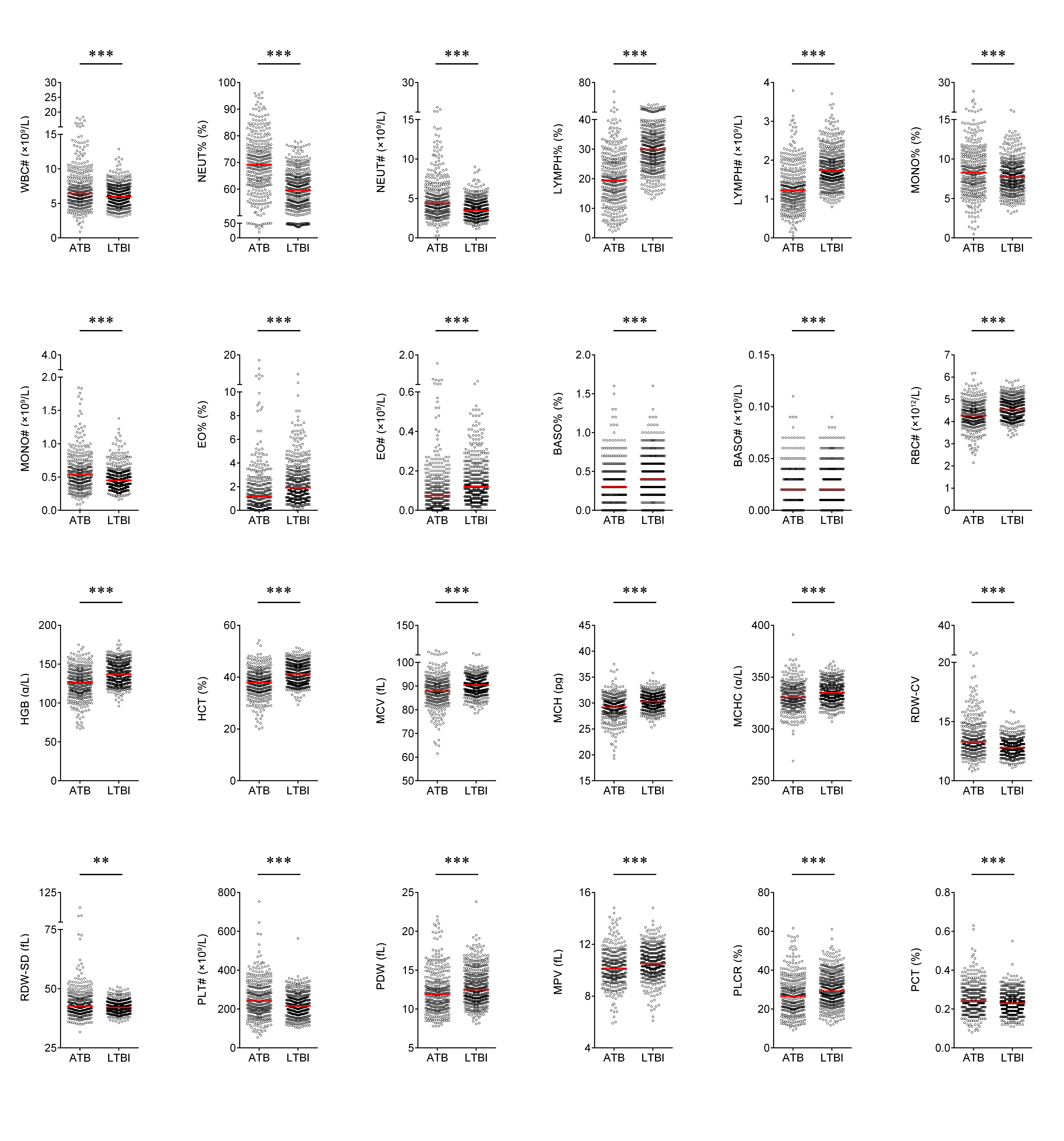

Supplement: Supplementary Figure 2 — The results of BRE in ATB patients (n = 372) and LTBI individuals (n = 511) in Caidian cohort. **P < 0.01, ***P < 0.001 (Mann-Whitney U test). WBC#, white blood cell count; NEUT%, neutrophil percentage; NEUT#, neutrophil count; LYMPH%, lymphocyte percentage; LYMPH#, lymphocyte count; MONO%, monocyte percentage; MONO#, monocyte count; EO%, eosinophil percentage; EO#, eosinophil count; BASO%, basophil percentage; BASO#, basophil count; RBC#, red blood cell count; HGB, hemoglobin; HCT, hematocrit; MCV, mean corpuscular volume; MCH, mean corpuscular hemoglobin; MCHC, mean corpuscular hemoglobin concentration; RDW-CV, coefficient variation of red blood cell volume distribution width; RDW-SD, standard deviation in red cell distribution width; PLT#, platelet count; PDW, platelet distribution width; MPV, mean platelet volume; PLCR, platelet larger cell ratio; PCT, thrombocytocrit. ATB, active tuberculosis; LTBI, latent tuberculosis infection; BRE, blood routine examination. [file Image_2.tif]
